# The Practitioner's Guide

**Published:** 1902-12

**Authors:** 


**The Practitioner's Guide.** By J. Walter Carr, M.D. Lond., F.R.C.P.; T. Pickering Pick, F.R.C.S.; Alban H. G. Doran, F.R.C.S.; and Andrew Duncan, M.D., B.S. Lond., F.R.C.S. Pp. vi., 1107. London: Longmans, Green & Co. 1902.

The authors have endeavoured, as far as possible, to make this book what its title suggests that it is—a Guide to the General Practitioner, and they have devoted the space at their command to a description of those subjects which they believed would be useful. It does not profess itself to be a complete Encyclopædia in one volume, but it certainly is a handy reference book for the busy man, and being in Dictionary form any topic is easily found. An index of seventeen pages also adds to its utility as a ready-reference book for consultation at odd minutes. The names of the authors are a sufficient guarantee of the reliable character of the work.

The value of such a book is no doubt mainly in the direction of refreshing the memory upon various little points forgotten since the time of reading more systematic works. It will give practical aid, and often also a concise summary of the more scientific aspects of disease. At the same time books of this character can rarely be considered to speak authoritatively on all points. There will be many who will differ from such a statement as that which says, "high arterial tension kept up by 'over-eating, over-drinking, and constipation,' is particularly apt to lead to adhesions of the cusps, and so to mitral stenosis." We should also differ from the view that the strain thrown upon the tricuspid valve in cases of emphysema of the lungs "may lead to the supervention of chronic and inflammatory changes" in the valve. Little blemishes of this character do not, however, seriously detract from the value of the work.

